# Mechano‐Nanoswitches for Ultrasound‐Controlled Drug Activation

**DOI:** 10.1002/advs.202104696

**Published:** 2022-02-23

**Authors:** Shuaidong Huo, Zhihuan Liao, Pengkun Zhao, Yu Zhou, Robert Göstl, Andreas Herrmann

**Affiliations:** ^1^ Fujian Provincial Key Laboratory of Innovative Drug Target Research School of Pharmaceutical Science Xiamen University Xiamen 361102 China; ^2^ DWI – Leibniz Institute for Interactive Materials Forckenbeckstr. 50 Aachen 52056 Germany; ^3^ Institute of Technical and Macromolecular Chemistry RWTH Aachen University Worringerweg 1 Aachen 52074 Germany

**Keywords:** dimer, drug activation, drug release, nanoswitch, ultrasound

## Abstract

Current pharmacotherapy is challenged by side effects and drug resistance issues due to the lack of drug selectivity. Mechanochemistry‐based strategies provide new avenues to overcome the related problems by improving drug selectivity. It is recently shown that sonomechanical bond scission enables the remote‐controlled drug release from their inactive parent macromolecules using ultrasound (US). To further expand the scope of the US‐controlled drug activation strategy, herein a mechano‐responsive nanoswitch for the selective activation of doxorubicin (DOX) to inhibit cancer cell proliferation is constructed. As a proof‐of‐concept, the synthesis, characterization, and US‐responsive drug activation evaluation of the mechano‐nanoswitch, which provides a blueprint for tailoring nanosystems for force‐induced pharmacotherapy is presented.

## Graphical abstract

Schematic representation of Au‐DNA dimer nanoswitches for ultrasound‐controlled activation of doxorubicin for selective cancer cell inhibition.

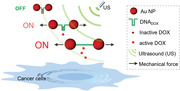

## 1 Introduction

Pharmaceutical drugs are undoubtedly the most crucial resource for cancer treatment. However, the widespread application of chemical drugs is compromised by their undesirable side effects, and patients usually suffer severely during long‐term chemotherapy.^[^ ^1^, ^2^, ^3^ ^]^ Therefore, advanced therapeutic strategies are urgently needed to increase drug efficacy and meantime decrease side effects.

In recent decades, therapeutic systems that can respond to specific stimuli, including internal (e.g., pH change,^[^ ^4^ ^]^ redox activities,^[^ ^5^ ^]^ etc.) or alternatively external triggers (e.g., light irradiation,^[^ ^6^ ^]^ electromagnetic fields,^[^ ^7^ ^]^ etc.), have been constructed to realize controlled release of therapeutic agents. Nevertheless, without control over the drug activity, these strategies still face the limitation of poor drug selectivity, premature drug leakage, low controllability, and others.^[^ ^8^, ^9^ ^]^ To this end, dynamic full‐control of drug activity is highly desirable, allowing remote activation of drugs at the site of action regardless of the selected target, thus resulting in more effective and precise treatment.

The emerging field of mechanochemistry provides new possibilities to alter drug activity.^[^ ^10^, ^11^, ^12^ ^]^ Compared with traditional thermal or photochemical reactions, simple mechanical force, such as ultrasound‐induced shear force, can induce chemical transformations by cleaving or rearranging bonds of force‐sensitive molecular motifs (mechanophore).^[^ ^13^, ^14^, ^15^, ^16^ ^]^ Moreover, the force‐induced molecular conversion can be easily regulated by tuning ultrasonication's frequency and exposure time.^[^ ^17^ ^]^ Recently, we provided the first example of US‐induced mechanochemical bond scission for the activation of drugs, underlining the potential of US for spatial and temporal regulation of medication.^[^ ^18^, ^19^ ^]^ Furthermore, the combination of nanoparticle systems with polymer mechanochemistry enabled increased drug loading efficiency and enhanced mechanical responses compared to systems relying exclusively on synthetic polymers.^[^ ^20^ ^]^

**Scheme 1:**
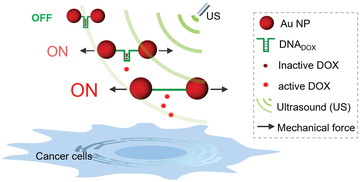
Schematic representation of Au‐DNA dimer nanoswitches for ultrasound‐controlled activation of DOX for selective cancer cell inhibition.

To further expand the scope of the US‐induced drug activation in nanosystems, herein we construct a mechano‐nanoswitch for the selective activation of anticancer drug doxorubicin (DOX) through ultrasonication. Using the principle of mechanochemistry, two gold nanoparticles (AuNPs) act as a transmitter of the shear force. Thereby, double‐stranded (ds) DNA was connected between two AuNPs and functions as a force‐sensitive unit (mechanophore). It is well known that the anticancer drug DOX contains flat aromatic rings and a free amino functional group, can preferentially intercalate into dsDNA 5′‐GC‐3′ or 5′‐CG‐3′ (DNA_DOX_) through noncovalent interactions, resulting in the deactivation and fluorescence‐quenching of the drug.^[^ ^21^ ^]^ Upon US irradiation, the nanoswitch loaded with DOX was stretched, and the specific noncovalent interactions between the drug and the DNA were cleaved, leading to the release and activation of DOX (**Scheme** **1**). Notably, the spatiotemporal control over drug activity could be well‐regulated by tuning the exposure time of ultrasonication during this process. We believe that this proof‐of‐concept approach is a significant step toward designing force‐induced nanosystems for precise drug activity regulation.

## 2 Results and Discussion

### 2.1 Preparation and Characterization of Au‐DNA Dimer Nanoswitches

**Figure 1:**
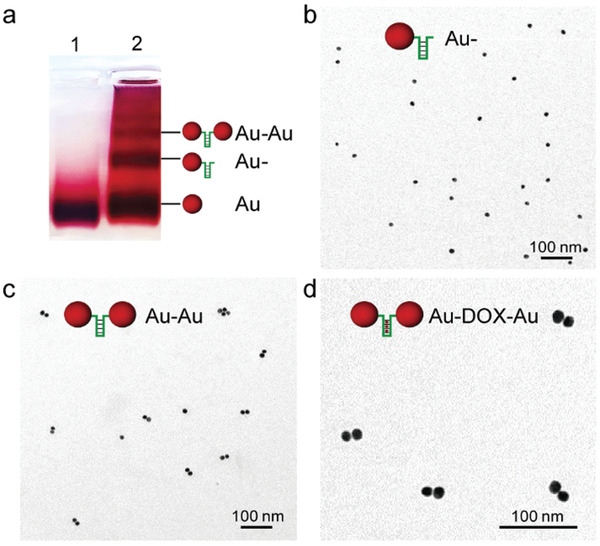
Purification and characterization of Au‐DNA dimer nanoswitches. a) Electrophoretic analysis of DNA‐bridged Au dimers formed from DNA_DOX_ sequences linked by 15 nm AuNPs. Lane 1: pristine AuNPs as a reference; Lane 2: separate bands from a reaction mixture of AuNP functionalized with terminally double‐thiolated DNA sequences. b) Representative TEM image of Au‐DNA_DOX_ (Au‐) purified by agarose gel electrophoresis. c) Representative TEM image of Au‐DNA_DOX_ dimer (Au–Au) purified by agarose gel electrophoresis. d) Representative TEM image of Au‐DNA_DOX_ dimer incubated with DOX (Au‐DOX‐Au).

We first synthesized citrate‐protected 15 nm gold nanoparticles (AuNPs) and then modified them with terminally double‐thiolated ssDNA (Table S1, Supporting Information) as reported before.^[^ ^22^ ^]^ The conjugation efficiency was determined by Image J (Figure S1, Supporting Information), and the Au‐DNA dimer bridged with a ssDNA strand was finally isolated and recovered from the reaction mixture by agarose gel electrophoresis. As shown in **Figure** **1**a, the separate bands display gradually slower mobility in the order of bare AuNPs (Au), single DNA modified AuNP conjugates (Au‐) and DNA‐bridged AuNP dimers (Au–Au). Transmission electron microscopy (TEM) images further proved the high purity of dimer production (Figure 1b,c). Statistical analysis of additional TEM images, obtained from the dimer band, allowed to estimate the yield of Au‐DNA dimer to be above 80%. It should be noted that the designed ssDNA sequence tends to form a hairpin structure containing 5′‐GC‐3′ repeats for DOX intercalation (Figure S2, Supporting Information), and the loading of DOX molecules induced no visible change to the closed dimer structure (Au‐DOX‐Au, Figure 1d).

### 2.2 Drug Payload Capacity of Au‐DNA Dimer Nanoswitches

**Figure 2:**
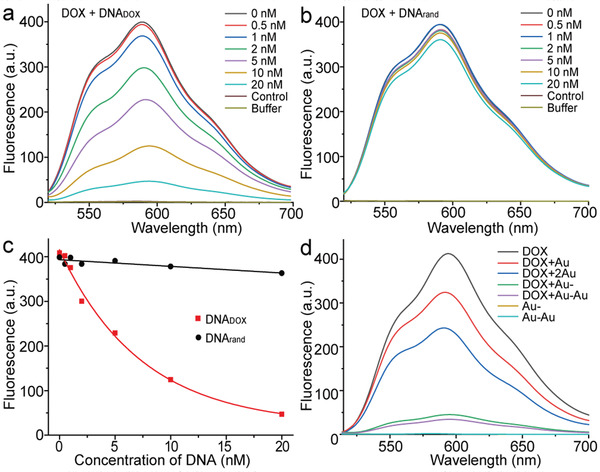
The drug payload capacity of Au‐DNA dimer nanoswitches. Fluorescence spectra (*E*_x_ = 488 nm) of DOX (100 × 10^−9^ m) with increasing equivalents of a) ssDNA with DOX intercalating sites (DNA_DOX_), and b) ssDNA with random sequence (DNA_rand_). c) The fluorescence quenching capacity analysis of the DNA_DOX_ compared with DNA_rand_. Data points show the fluorescence intensities (*E*_m_ = 591 nm) at varying concentrations of DNA. Data were fit by a nonlinear regression model with solid lines. d) Fluorescence spectra of DOX (100 × 10^−9^ m) upon the addition of AuNP, AuNP (2 × ), Au‐, and Au–Au, respectively. Equivalents of Au‐ and Au–Au were tested as controls.

The Au‐DNA dimer architecture was then evaluated concerning drug loading. It was previously noted that the DNA_DOX_ bridge between the two nanoparticles provides many spatially addressable sites for drug loading. Then the DOX loading of Au–Au by intercalation was investigated by fluorescence spectroscopy. Due to the fact that each ssDNA_DOX_ sequence theoretically harbors about ten drug loading sites (Figure S2, Supporting Information), the fluorescence of DOX is gradually quenched with increasing addition of the DNA_DOX_ sequence (**Figure** **2**a). The addition of 10 × 10^−9^ m DNA_DOX_ led to ≈70% decrease of the initial fluorescence intensity. As a control, the addition of ssDNA with random sequence (DNA_rand_) showed no significant quenching effect of DOX molecules (Figure 2b), demonstrating the selective DOX intercalation capacity of DNA_DOX_ (Figure 2c). In principle, the drug payload capacity of the DNA_DOX_ sequence could be increased and tailored by changing the sequence length and number of drug intercalation sites.

Afterward, the drug loading capacity of Au–Au was further proved and quantified using the same methods (Figure 2d). The obtained fluorescence spectra verified the drug payload capacity of the Au–Au nanoswitches, and the drug loading efficiency was calculated at ≈64%, according to the formula shown in supporting information. It is worth noting that, due to the surface plasmon resonance of gold nanoparticles, the mixture of DOX with naked AuNP shows a certain degree of fluorescence quenching, which has a negligible impact on this study.

### 2.3 US‐Induced Structural Change of Au‐DOX‐Au Dimer Nanoswitches

**Figure 3:**
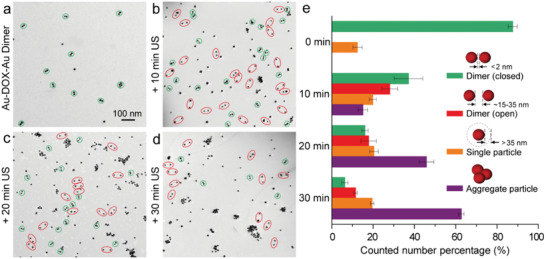
US‐induced structural change of Au‐DOX‐Au dimer nanoswitches. Representative TEM images of a) Au‐DOX‐Au without ultrasonication, Au‐DOX‐Au with ultrasonication for b) 10 min, c) 20 min, and d) 30 min, respectively (green circled: closed; red circled: open). e) The corresponding number percentage histograms of Au‐DOX‐Au with different morphologies after US treatment for 0, 10, 20, and 30 min, respectively. The statistical analysis was repeated with three independent samples. Mean values: SD from the mean, *N* = 3 independent experiments.

To verify our proposed hypothesis of applying mechanical force to stretch the Au‐DNA dimer structures to achieve drug release, we studied the US responsiveness of the Au‐DOX‐Au system upon ultrasonication. With the help of TEM, we found that part of the initially closed dimer (green circled in **Figure** **3**a) changed to an open state with a sizeable interparticle gap (≈15–35 nm) after 10 min ultrasonication (red circled in Figure 3b), showing the force‐response ability of the dimer structure. This change could be explained by the US‐induced dissociation of the base‐pair interactions within the Au‐DNA dimer structure. With ultrasonication for a longer time, the number of closed dimers continually decreased, indicating more dimer structures were force‐stretched (Figure 3c,d). Meanwhile, an increasing number of single‐particle and aggregate formations was monitored over the course of sonication. This observation is most likely caused by covalent bond scission along the ssDNA backbone with long‐time ultrasonication. In addition to the TEM observation, we characterized the size changes of Au–Au dimers before and after ultrasonication (Figure S3, Supporting Information). Upon the sonication, the dynamic size distribution of dimer (centered at ≈40 nm) shifted to ≈100 nm and gradually started to generate large aggregates, which is generally consistent with the TEM observation results.

For a more accurate study, several TEM images were recorded, measured, and statistically analyzed during the ultrasonication process.^[^ ^23^ ^]^ As shown in Figure 3e, the number percentage of the closed dimer was above 80% initially, while it decreased to ≈40% after 10 min US treatment and only ≈10 % left after 30 min ultrasonication, suggesting the high efficiency of US‐induced mechanical stretching of the Au‐DNA dimer structure. At the same time, the number of the dimer with the open state was found to increase first and then decrease, accompanied by the appearance of single‐particles and aggregates, indicating that the continued ultrasonication further destroyed particle surface functionalization and their colloidal stability.

### 2.4 US‐Induced Drug Release and Activation of Nanoswitches

**Figure 4:**
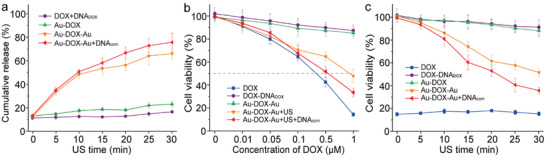
US‐controlled drug release and activation inhibiting cancer cell proliferation. a) The calculated cumulative release of DOX from DNA_DOX_, Au‐DOX, Au‐DOX‐Au dimer, and Au‐DOX‐Au dimer + DNA_com_ in response to ultrasonication for different times, respectively. The experiments were carried out in triplicate. Mean values: SD from the mean, *N* = 3 independent experiments. b) Cell proliferation assay involving LNCaP cells with different concentrations of free DOX, DOX+DNA_DOX_, Au‐DOX‐Au dimer without or with 30 min ex situ ultrasonication, and Au‐DOX‐Au dimer + DNA_com_ with 30 min ex situ ultrasonication, respectively. The experiments were performed in triplicate. Mean values: SD from the mean, *N* = 3 independent experiments. c) Cell proliferation assay involving LNCaP cells with equivalents of 1 × 10^−6^ m free DOX, DOX+DNA_DOX_, Au‐DOX NP, Au‐DOX‐Au dimer, and Au‐DOX‐Au dimer + DNA_com_ against ex situ ultrasonication for different times, respectively. The experiments were carried out in triplicate. Mean values: SD from the mean, *N* = 3 independent experiments.

Having demonstrated the force‐induced stretching behavior of the Au‐DOX‐Au dimer nanoswitches, the drug release and inhibition of cancer cell proliferation were determined. Before that, we first verified the activation capability of the system by the turn‐on fluorescence of DOX upon different periods of ultrasonication. As shown in **Figure** **4**a, a 30 min irradiation of Au‐DOX‐Au with US in solution led to ≈60% fluorescence recovery, indicating about 60% of DOX was released from the nanoswitches. To prevent the released DOX molecules from recombining with the structure, a complementary ssDNA (DNA_com_) for DNA_DOX_ was added during the course of sonication. Since the addition of DNA_com_ could competitively hybridize with DNA_DOX_ after DOX release, meantime, long‐time ultrasonication could destroy the Au‐DNA‐Au structure, resulting in rare binding sites for DOX recombination. As a result, the drug release percentage was obviously (≈70%) than the corresponding group without DNA_com_ after 30 min ultrasonication (≈63%). In contrast, the control groups (DOX+DNA_DOX_ and Au‐DOX NP) did not lead to an increase in fluorescence under the same conditions, demonstrating the mechanochemical origin of the nanoswitches’ activation.

We then investigated the activity of US‐responsive nanoswitches in comparison to a series of controls through an 3‐(4,5)‐dimethylthiahiazo (‐z‐y1)‐3,5‐diphenytetrazoliumromide (MTT) cell proliferation assay (Figure 4b). The considerably higher half‐maximal inhibitory concentration (IC50) values of DOX+DNA_DOX_ and Au‐DOX‐Au compared with DOX clearly demonstrated that DOX intercalation within DNA_DOX_ decreased the drug activity, and at the same time, indicated the excellent biocompatibility of the Au‐DNA dimer platform. Since LNCaP cells were not viable under prolonged sonication conditions, we subjected the samples to 30 min US irradiation ex situ before mixing them with cell culture medium to investigate the activity of nanoswitches upon US irradiation. As a result, the decrease in cell viability with ultrasonication is apparent and indicates the successful sonomechanical activation of Au‐DOX‐Au. Notably, the addition of DNA_com_ further enhanced the US‐induced drug activation, which is consistent with the result above. To further verify the US‐controlled drug activation process, LNCaP cells were incubated with Au‐DOX‐Au after different ultrasonication periods. As shown in Figure 4c, the results demonstrated that the drug activity of nanoswitches could be spatiotemporally controlled by regulating the exposure time of ultrasonication.

## 3 Conclusion

In summary, for the first time, we exploited ultrasound as an external stimulus for spatiotemporal controlled drug release and activation using a sonomechanical responsive Au‐DNA nanoswitch. Our design is based on the facile functionalization of nanoparticles and drug intercalation into the DNA structure omitting laborious chemical synthesis. The nanoswitch system proved to be US‐responsive and could be used for mechanically controlled drug activation to realize selective inhibition of cancer cell proliferation. Theoretically, the US‐activated nanoswitches proposed in this work are universally applicable to other drug‐sequence complexes. Meanwhile, one of the most longstanding challenges for transferring mechanochemistry to therapeutic ultrasound applications is to find a window of opportunity to activate the desired compounds while minimizing unwanted tissue damage.^[^ ^24^ ^]^ We believe this proof‐of‐concept approach provides a blueprint for constructing drug activation nanoparticle systems with external control of ultrasound. With this approach, we conceived of a future pharmacotherapy that could have a well‐regulated activity that might avoid systemic side effects.

## Conflict of Interest

The authors declare no conflict of interest.

## Supporting information

Supporting Information

## Data Availability

The data that support the findings of this study are available from the corresponding author upon reasonable request.
