# Supplementary material for: Mechano‐Nanoswitches for Ultrasound‐Controlled Drug Activation
Source: Adv Sci (Weinh). 2022 Feb 23;9(12):2104696. doi: 10.1002/advs.202104696 (PMC9036040; doi:10.1002/advs.202104696)
Supplement: Supplementary file 1 — Supporting Information [file ADVS-9-2104696-s001.pdf]

ADVANCED SCIENCE
Open Access

Supporting Information

for *Adv. Sci.*, DOI 10.1002/advs.202104696

Mechano-Nanoswitches for Ultrasound-Controlled Drug Activation

Shuaidong Huo, Zhihuan Liao, Pengkun Zhao, Yu Zhou, Robert Göstl and Andreas Herrmann**

Electronic Supplementary Information

for

Mechano-Nanoswitches for Ultrasound-Controlled Drug Activation

Shuaidong Huo,[a,b,c,*] Zhihuan Liao,[a] Pengkun Zhao,[b] Yu Zhou,[b] Robert Göstl [b] and Andreas Herrmann [b,c,*]

[a]Fujian Provincial Key Laboratory of Innovative Drug Target Research, School of Pharmaceutical Science, Xiamen University, 361102 Xiamen, China

[b]DWI – Leibniz Institute for Interactive Materials, Forckenbeckstr. 50, 52056 Aachen, Germany

[c]Institute of Technical and Macromolecular Chemistry, RWTH Aachen University, Worringerweg 1, 52074 Aachen, Germany

*Correspondence to: huosd@xmu.edu.cn and herrmann@dwi.rwth-aachen.de

1. Materials

All chemical reagents were of analytical grade and were used without further purification if not stated otherwise. Chloroauric acid ($HAuCl_4$), sodium citrate, bis(p-sulfonatophenyl)phenylphosphine dehydrate dipotassium (BSPP), sodium borohydride, tris(2-carboxyethyl) phosphine hydrochloride (TCEP·HCl), Doxorubicin hydrochloride (DOX·HCl) and agarose were obtained from Sigma-Aldrich and used as received. Other chemicals were purchased from Sigma-Aldrich unless otherwise noted. Milli-Q water was used throughout all the experiments. All the oligonucleotide (ODN) sequences listed in Table S1 were synthesized, HPLC-purified, and verified by MALDI-ToF mass spectrometry by Biomers Co. Ltd. (Germany).

2. Methods

Preparation of Au-DNA dimer nanoswitches (Au-Au)

15 nm Au-Citrate NPs were prepared by the standard citrate reduction method.[1] In brief, 4 mL of 25 mM $HAuCl_4$ solution in 96 mL of Milli-Q water was heated to reflux, and then 10 mL of 38.8 mM sodium citrate solution was added to the boiling solution quickly under vigorous stirring. After a color change was observed, the mixture was kept boiling for another 30 min and then cooled down to room temperature with gentle stirring. To the obtained 15 nm citrate-stabilized AuNPs, BSPP salt was added to realize surface ligand exchange as reported.[2] Au-DNA conjugates were prepared by mixing Au NPs with the terminally double-thiolated ssDNA (TCEP treated) in a molar ratio of 3:1, and the mixture was incubated for 12 h in 0.5×TBE buffer (containing 50 mM NaCl). Finally, the Au-DNA dimer was isolated by extraction from a gel after gel electrophoresis,[3] and the conjugation efficiency was determined by Image J (Figure S1).

Agarose gel electrophoresis

3% agarose gel was prepared and used to determine the formation AuNP DNA conjugates as well as to isolate the AuNP dimer structure. The electrophoresis was performed as follows: 15 μL Au-DNA conjugates were mixed with 5 μL glycerin and

loaded onto an agarose gel gently. Electrophoresis was carried out at a voltage of 130 V for 30 min in 0.5×TBE running buffer. Finally, the AuNP-DNA conjugate dimer band was cut and recovered by a D-Tube™ electroelution accessory kit (D-Tube™ Dialyzer Midi) through another gel electrophoresis.

Transmission electron microscopy (TEM) observation and analysis

Samples were prepared and placed on a copper grid, and dried at room temperature. TEM images were examined and imaged using a Libra 120 Transmission Electron Microscope (Carl Zeiss) with 120 kV accelerating voltage.

For the interparticle distance study, several TEM images were recorded, measured, and statistically analyzed after different ultrasonication times. The distances of each particle with its adjacent particles were measured and recorded. The nanoparticle distance between each other of the dimer is below 2 nm. Since the theoretical length of double-thiolated ssDNA (DNA_{DOX}) is calculated at ca. 35 nm, we use 35 nm as the critical size to determine the dimer (open) and single particle. The observation and measurement were repeated with three independent samples.

Hydrodynamic diameter measurement and analysis

The hydrodynamic diameter of the prepared Au NPs and Au-DNA dimer structures before and after ultrasonication were determined by a Nano ZS Zetasizer (25 °C, Malvern, England).

DNA annealing and doxorubicin intercalation

The DNA sequence was dissolved in annealing buffer (200 mM KCl, 4 mM $MgCl_2$, and 28 mM Tris-HCl) and TE buffer (10 mM Tris-HCl, 1 mM EDTA, and 100 mM NaCl), respectively, and then annealed at 100°C for 5 min. Then the mixture was cooled slowly to room temperature to form a double-stranded DNA structure (closed configuration).

For the doxorubicin intercalation, different amount of doxorubicin was incubated with the Au-DNA dimer in an ice bath and shaken gently in the dark for 1 h. After

that, the complex was centrifuged to remove excess free doxorubicin. The DOX loading efficiency was caculated based on the following formula:

Doxorubicin loading effiency (%) = [1- $(Fluo_{free}-Fluo_{buffer})/(Fluo_{total}-Fluo_{buffer})$] × 100%

Fluorescence assays

The drug loading and drug release were monitored by fluorescence spectroscopy. Samples were measured with a spectrophotometer (SpectraMax M3, Molecular Devices) under an excitation wavelength of λ = 488 nm at 25 °C.

For studying the mechanochemical response of the drug release, each sample was centrifuged, and the fluorescence emission intensity of the supernatant was measured at λ = 591 nm immediately after ultrasonication, under an excitation wavelength λ = 488 nm at 25 °C.

Sonication experiments

Ultrasonication experiments on the samples were performed in a 1 mL ultrasonication vessel (Test tube heavy-walled, 2775/2, Assistant) with a Qsonica Q125 sonicator (USA) equipped with a 3 mm diameter microtip probe (A12628PRB20). Sonication was performed using pulsed ultrasound (1.0 s on, 1.0 s off at 50% Amplitude) at f = 20 kHz. The vessel was placed in an ice bath to maintain a temperature inside the vessel of 6-9 °C throughout sonication.

Cell Culture

Human prostate cancer LNCaP cells were obtained from the American Type Culture Collection (ATCC). Cells were cultured in RPMI medium 1640 supplemented with 10% Fetal Bovine Serum (FBS) and 100 IU/mL penicillin-streptomycin at 37 °C in a humid atmosphere water-jacketed incubator with 5% CO_2.

Cell cytotoxicity assay

The cytotoxicity of LNCaP cells to different sample treatments was determined by MTT (3-(4,5)-dimethylthiahiazo (-z-y1)-3,5- diphenytetrazoliumromide) assay. Briefly, 100 μL culture medium containing cells at a suitable density were plated in 96-well plates. After incubation for 24 h, the cells were treated with free DOX, DOX+DNA_{DOX}, Au-DOX-Au dimer without or with 30 min ex situ ultrasonication, and Au-DOX-Au dimer + DNA_{com} with 30 min ex situ ultrasonication for 48 h, respectively. Then the medium was replaced with 100 mL MTT (0.5 mg/mL) and further cultured for another 3 h. Subsequently, the MTT solution was replaced with 150 μL of dimethyl sulfoxide solution. Finally, the absorbance at 570 nm of each well was measured using a microtiter plate reader. Untreated cells in the medium were used as a control. For the cell viability experiments over the course of sonication, cells were incubated with ultrasonication samples (free DOX, DOX+DNA_{DOX}, Au-DOX, Au-DOX-Au dimer, and Au-DOX-Au dimer + DNA_{com}) for 48 h, respectively. The ultrasonication period was 0, 5, 10, 15, 20, 25, 30 min, respectively. All SDs were calculated from three replicates

Statistical analysis

All experiments were carried out in triplicate unless otherwise indicated. Error bars represent standard deviations. Data are presented as mean value ± SD from three independent measurements. Analysis was performed using Origin software (version: 2018, OriginLab Inc., USA). The number of samples for each analysis was introduced in each figure legend.

3. Supplemental figures

Table S1. DNA sequences used in this work.

DNA name	Sequences
1. *DNA$_{DOX}$	SH-5'-AAAAAAAAAAAAAAAAAAAAAGGA GGAGGAGGAGGAAAAAATCCTCCTCCTC CTCCAAAAAAAAAAAAAAAAAAAAA-3'-SH
2. DNA$_{rand}$	SH-5'-AAAAAAAAAAAAAAAAAAAAAAGGA GGAGGAGGAGGAAAAAAGGAGGAGGAG GAGGAAAAAAAAAAAAAAAAAAAAAA-3'-SH
3.**DNA$_{com}$	3'TTTTTTTTTTTTTTTTTTTTTTTTTTTA5'
*The DOX molecules can preferentially intercalate into double-stranded 5′-GC-3′ or 5′-CG-3′. **DNA$_{com}$ is partly hybridized with DNA$_{DOX}$ to prevent the formation of hairpin structure for DOX intercalation.	

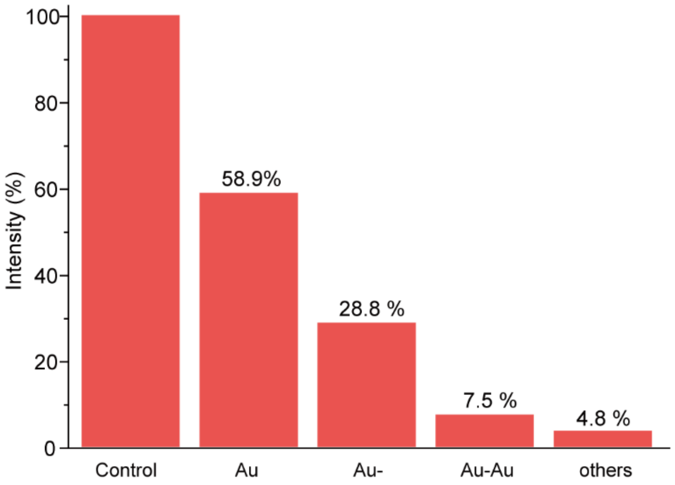

Figure S1. The production efficiency of each component after Au-DNA conjugation reaction. The intensity was quantified by ImageJ from the gel image in Figure 1a.

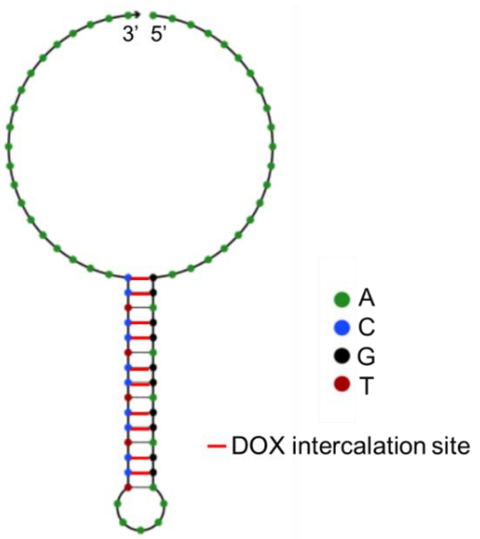

Figure S2. Predicted secondary structures of DNA_{DOX} sequence for DOX intercalation. Structures were predicted using the Nupack software at 37 ℃.

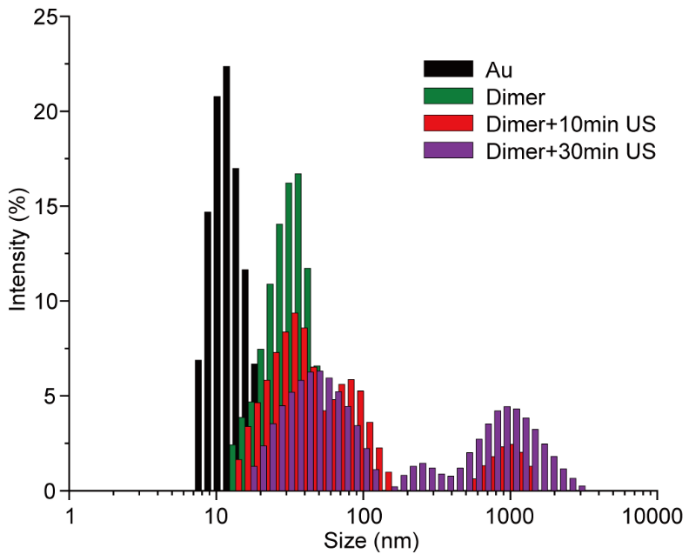

Figure S3. The hydrodynamic size analysis of naked Au NP, Au-DNA dimer, and Au-DNA dimer with ultrasonication for 10 min, 30 min, respectively.

4. References

1. Liu, J., and Lu, Y. *Nat. Protoc.*, 2006, **1**, 246-252.
2. Wang, H., Li, Y., Liu, M., Gong, M. and Deng, Z. *Small*, 2015, **11**, 2247-2251.
3. Li Y, Cheng Y, Xu L, Du H, Zhang P, Wen Y, and Zhang X. *Nanomaterials*. 2016, **6**, 24.
